# Rural-urban differences in the mental health of perinatal women: a UK-based cross-sectional study

**DOI:** 10.1186/s12884-020-03132-2

**Published:** 2020-08-14

**Authors:** Samuel Ginja, Katherine Jackson, James J. Newham, Emily J. Henderson, Debbie Smart, Raghu Lingam

**Affiliations:** 1 School of Psychology, Ulster University, Cromore Road, Coleraine, BT52 1SA Northern Ireland, UK; 2 Department of Sociology, Durham University, 29 Old Elvet, Durham, DH1 3HN England, UK; 3 Department of Psychology, Faculty of Health and Life Sciences, Northumberland Building, Northumbria University, Newcastle upon Tyne, NE1 8ST England, UK; 4 Children & Young People’s Mental Health & Wellbeing, Newcastle upon Tyne, NE7 7XA England, UK; 5 Social Work, Education & Community Wellbeing, Northumbria University, Newcastle upon Tyne, NE7 7XA England, UK; 6 Population Health Sciences Institute, Faculty of Medical Sciences, Newcastle University, Newcastle upon Tyne, NE2 4AX England, UK; 7 Population Child Health Clinical Research Group, School of Women’s and Children’s Health, Faculty of Medicine, University of New South Wales, Rm 814, Level 8 The Bright Alliance, High St & Avoca Street, Randwick, NSW 2031 Australia

**Keywords:** Rural, Urban, Mental health, Perinatal, Antenatal, Postnatal

## Abstract

**Background:**

International data suggest that living in a rural area is associated with an increased risk of perinatal mental illness. This study tested the association between rurality and risk for two mental illnesses prevalent in perinatal women - depression and anxiety.

**Methods:**

Using a cross-sectional design, antenatal and postnatal women were approached by healthcare professionals and through other networks in a county in Northern England (UK). After providing informed consent, women completed a questionnaire where they indicated their postcode (used to determine rural-urban status) and completed three outcome measures: the Edinburgh Postnatal Depression Scale (EPDS), the Whooley questions (depression measure), and the Generalised Anxiety Disorder 2-item (GAD-2). Logistic regression models were developed, both unadjusted and adjusted for potential confounders, including socioeconomic status, social support and perinatal stage.

**Results:**

Two hundred ninety-five participants provided valid data. Women in rural areas (*n* = 130) were mostly comparable to their urban counterparts (*n* = 165). Risk for depression and/or anxiety was found to be higher in the rural group across all models: unadjusted OR 1.67 (0.42) 95% CI 1.03 to 2.72, *p* = .038. This difference though indicative did not reach statistical significance after adjusting for socioeconomic status and perinatal stage (OR 1.57 (0.40), 95% CI 0.95 to 2.58, *p* = .078), and for social support (OR 1.65 (0.46), 95% CI 0.96 to 2.84, *p* = .070).

**Conclusions:**

Data suggested that women in rural areas were at higher risk of depression and anxiety than their urban counterparts. Further work should be undertaken to corroborate these findings and investigate the underlying factors. This will help inform future interventions and the allocation of perinatal services to where they are most needed.

## Background

Mental illness, most commonly depression and anxiety, affects up to 20% of women in the UK during pregnancy and in the first year after childbirth [1]. Understanding the reasons for this problem requires consideration of the social, economic and geographical environments in which mothers live, which can greatly differ within a given country. International data, including from high-income, middle-income and low-income countries, suggest that perinatal mental illness is more prevalent in rural women compared to their urban counterparts and to the general population [2, 3]. Other negative outcomes have been reported to be more frequent in women from rural areas including later initiation of prenatal care [4], later and less use of contraceptives [5], as well as higher chances of a small-for-gestational-age birth and low birth-weight [6], all of which may add to stress levels and increase the risk of mental illness. Possible mediators for these rural-urban differences include difficulties in accessing healthcare services, regional variations in cultural practices, sociodemographic and lifestyle factors [3, 6, 7], as well as varying levels of hazardous environmental, occupational and transportation conditions [8]. Social support and socioeconomic status are particularly important because they are related to many of the above factors [3, 9, 10].

In the UK context, rural-urban status has been found to be associated with behavioural and emotional problems in children, although this was explained by the quality of their schools [11]. Also relevant is the finding that in the UK increased neighbourhood deprivation has been found to predict poor maternal mental health outcomes [12], particularly amongst older mothers [13], and that regional differences in the rate of referrals to perinatal mental health services can vary up to 20% [14]. However, to the best of our knowledge no study has looked specifically at the association between rural-urban status and maternal mental health outcomes in the country, and much of the existing literature is based on exclusively rural or urban samples which hinders comparisons between the two groups.

The aim of the present study was to assess the association between rurality (i.e., living in a rural area) and the risk for two common mental illnesses (depression and anxiety) during the antenatal and postnatal periods, i.e. perinatal period, in women from a county in Northern England (UK).

## Methods

### Study design

A cross-sectional survey in the form of an online and paper-based questionnaire was carried out between February and August 2017. Our target sample was based on existing data that suggested up to 20% regional variation in the rates of admission to perinatal services [14]. As this was an upper limit, we adopted a more conservative criterion of 15% difference between rural (lower) and urban (higher) groups. Our sample size calculation indicated that 348 participants would be needed to detect such difference in the odds of being at risk for mental illness, assuming a 5% alpha level, a 90% power, and a rural-urban ratio of one to one.

### Ethics

This study was given ethical approval by the Newcastle & North Tyneside 1 Research Ethics Committee (212364) and by the governance framework of the children and young people’s service in the county council area where the study took place.

### Participants

Participants were eligible if they were a) women aged 16 years or above, b) pregnant (at any stage of gestation) or who had had a baby in the last 12 months prior to enrolment into the study, c) able to provide informed consent in English. Due to limited resources, it was not possible to translate materials into other languages. This work was undertaken as part of a wider project aimed at exploring the needs and experiences of women in rural communities in the area, relating to their mental health during and after pregnancy, which involved both quantitative and qualitative research.

### Recruitment

Some participants were informed about the study by their health visitor antenatally (28–34 weeks’ gestation) or postnatally (6 weeks post-delivery). In the UK, health visitors are qualified nurses or midwives working with antenatal and postnatal families offering support and advice, both through home visits and at medical centres [15]. Health visitors provided women with the study participant information sheet. Those who chose to take part and gave informed consent completed a questionnaire either during or after their health visitor’s home visit, either online or on paper. Completed paper questionnaires were returned to the research team in a sealed envelope through the health visitor or by post. Other women were recruited at the 20-week antenatal scan appointment, or at postnatal appointments, at one of three participating maternity hospitals in the region, or through social media (e.g. Facebook), local parenting groups, community midwives, or word of mouth. Participants recruited through these secondary methods received a flyer with information about the study and on how to access the online questionnaire. Online questionnaires were completed by participants after providing informed consent. All women had the option to contact the research team for any questions.

### Measures

The questionnaire (Additional file 1) was piloted for clarity in a sample of 10 women. Questions collected information on the exposure variable (rural vs urban areas), outcome variables (depression and anxiety) and covariates (sociodemographic information and social support). All variables were based on self-report, except rurality and socioeconomic deprivation.

#### Rurality

The exposure variable was the rural or urban status of the area in which participants lived. Participants were requested to provide their postcode, which was then used to determine their rural-urban status according to the following official 10-category classification [16]: 1) (most urban) urban major conurbation; 2) urban minor conurbation; 3) urban city and town; 4) urban city and town in a sparse setting; 5) rural town and fringe; 6) rural town and fringe in a sparse setting; 7) rural village; 8) rural village in a sparse setting; 9) rural hamlets and isolated dwellings; 10) (most rural) rural hamlets and isolated dwellings in a sparse setting. In this classification, a broader binary distinction between urban and rural areas is possible: an urban area is any between category 1 and category 4; a rural area is any between category 5 and category 10, which refer to settlements below 10,000 people or which are open countryside. The two-level broad classification was used for the main analyses, as any analysis based on a larger number of rural-urban categories would have resulted in small numbers in each category considering our sample size, which would have reduced the power of the analyses. An exploratory analysis was conducted that compared women in the two most rural categories to the two most urban categories to get the clearest picture of the difference between rural and urban status.

#### Markers of mental health

We used the Edinburgh Postnatal Depression Scale (EPDS) and the Whooley questions to measure risk of depression, and the 2-item General Anxiety Disorder Scale (GAD-2) to measure risk of anxiety. As part of routine care in the UK, these measures are used by healthcare professionals for screening for mental illnesses in perinatal women [17, 18].

The Edinburgh Postnatal Depression Scale (EPDS) has been used extensively both in antenatal and postnatal care [19], and has shown to be a valid and reliable tool across a large number of populations and contexts [20]. The scale comprises 10 items related to maternal feelings during the past 7 days, assessing depressed mood, guilt, anxiety, and suicidal ideation (e.g. “I have felt sad or miserable”). Items are given a score of 0 (lowest frequency or intensity) to 3 (highest frequency or intensity) and then summed to provide a total score, ranging from 0 to 30. A higher score reflects a higher level of depression. The recommended cut-off points for ‘probable major depression’ is 15 (for antenatal women) and 13 (for postnatal women); for ‘at least probable minor depression’, the threshold is 13 (for antenatal women) and 10 (for postnatal women) [19]. The latter criterion was adopted in the present study as we did not diagnose or refer women to clinical services, but rather assess how many women were at risk of (minor) depression. This also allowed us to maximise the number of participants in the analysis so that clearer conclusions could be drawn.

The Whooley questions consist of two ‘yes’ or ‘no’ questions about depressed mood (e.g. “During the past month, have you often been bothered by feeling down, depressed or hopeless?”) [21]. Answering yes to one or both questions indicates a positive screen. Common in routine clinical work, the Whooley questions have high sensitivity in detecting depression and should be used as a pre-diagnostic tool, in combination with other screening tools [22].

The 2-item General Anxiety Disorder Scale (GAD-2) is used to assess the frequency of symptoms of anxiety (e.g. “Over the last 2 weeks, how often have you been bothered by feeling nervous, anxious, or on edge?”) [23]. Respondents indicate how frequent the symptoms are on a Likert-scale from 0 (Not at all) to 3 (Nearly every day). This short scale is well-established in primary care and has shown high specificity to detect a range of anxiety disorders. Scores can vary from 0 to 6; a score of 3 or more indicates a positive screen [23].

We report these three measures separately, as well as combined into a binary measure, i.e., testing positive on one or more of the three measures. As none of the three tools used was intended to be diagnostic, we refer to risk of depression or anxiety in this study, rather than to rates of depression or anxiety.

#### Covariates

Data on two covariate variables - socioeconomic status and social support – were collected as these could potentially explain the association between rurality and outcomes, according to previous research [3, 9]. Socioeconomic status was based on the index of multiple deprivation (IMD) decile, a common indicator of affluence in the UK, which was determined by searching participants’ postcodes on an online tool [24].

Perceived social support was measured by the Multidimensional Scale of Perceived Social Support (MSPSS) [25] and considered as a potential mediating factor on the causal pathway between rurality and perinatal anxiety and depression. This scale consists of 12 statements about the support received from family (4 items), friends (4 items) and a significant other (4 items), e.g. ‘My family really tries to help me’. Participants rated their level of agreement with each statement on a seven-item Likert scale. Item scores were summed to provide total scores, both overall (range 12–84) and for each of the three subscales (range 4–28). Higher scores indicated perception of greater social support. The factorial validity and internal reliability of the MSPSS have been demonstrated in a number of studies, with alpha scores from 0.87 to 0.93 [25–27], including in pregnant women (alpha 0.92) [28].

Data on other variables was also collected, but these were only controlled for in the analysis if they differed between rural and urban groups. Those variables were age, perinatal stage (antenatal or postnatal), ethnicity, education, employment and relationship status. The potential role of these factors in maternal mental health outcomes has been reported in previous studies [29, 30]. Questions were adapted from previous Census surveys [31]. Additional questions asked participants about healthcare and community services, including mental health support.

### Data analysis

Questionnaire data were entered directly by participants using an online survey platform [32]; data from returned paper-based questionnaires were entered on to the platform by the research team. All analysis was completed in Stata 14 [33]. For overall scores consisting of sums, participants with one or more items missing were excluded as required by the instrument guidelines. Descriptive statistics and difference tests were performed to characterise the sample. When data were not normally distributed, the median and interquartile range (IQR) were reported.

There was a statistically significant difference in terms of perinatal stage between rural and urban groups; this was the only variable that differed significantly between the two groups. Consequently, perinatal stage was adjusted for in the main analysis (i.e., in addition to socioeconomic status and social support, which were purposively selected).

Multiple logistic regression models were developed in three steps to assess: a) the unadjusted association between rurality and depression and/or anxiety, b) this association adjusting for socioeconomic status and perinatal stage and c) the effect of social support on this adjusted association. The three-step process was used for each of the three outcomes independently. For each model, we reported the odds ratio for depression and/or anxiety associated with living in a rural area, together with the 95% confidence interval and significance level.

## Results

**Fig. 1:**
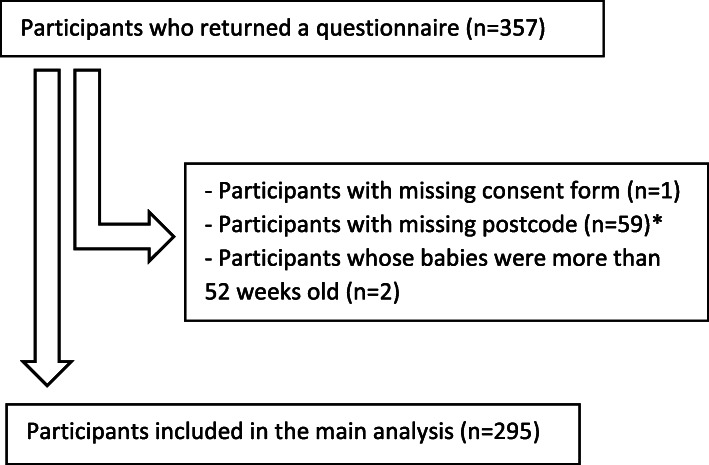
Participant flow diagram. *These 59 included participants with missing EPDS data (*n* = 21), missing Whooley data (*n* = 21), and missing GAD-2 data (*n* = 22)

Participant flow is shown in Fig. 1. A total of 295 participants were entered in the main analysis, all of whom had provided valid outcome data on the three measures and a valid postcode. Excluded participants did not differ significantly from others in age and perinatal stage; whether they differed in terms of IMD or urban/rural status cannot be determined due to missing postcodes. Approximately half of women heard about the study during a health visitor’s home visits (*n* = 137, 46.9%), others in antenatal or postnatal appointments at the hospital (*n* = 96, 32.9%), social media (*n* = 55, 18.8%), parenting groups (*n* = 3, 1.0%) or through other channels (*n* = 1, 0.3%).

The characteristics of study participants are presented in Table 1, by urban (*n* = 165, 55.9%) and rural (*n* = 130, 44.1%) groups, and overall (*N* = 295). There were no significant differences between urban and rural groups except for the perinatal stage; those from urban areas, compared to those from rural areas, were more likely to be currently pregnant (60.0% vs 47.7%, *p* = .035). Of those women who were pregnant, the median number of gestational weeks was 28 (IQR 15); of the postnatal women, the median number of weeks of the baby was 7 (IQR 12).

**Table 1:** Characteristics of participants (*N* = 295)

Variable	Missing (n)	Urban (*N* = 165), n (%)	Rural (*N* = 130), n (%)	Overall (*N* = 295), n (%)	Differences between groups
Age	0				
24 years or below		23 (13.9%)	11 (8.5%)	34 (11.5%)	X^2^ (4)=6.3, *p* = .179
25 years or above		142 (86.1%)	119 (91.5%)	261 (88.5%)	
Perinatal stage	0				
Pregnant		99 (60.0%)	62 (47.7%)	161 (54.6%)	X^2^ (1)=4.4, *p* = .035*
Postnatal		66 (40.0%)	68 (52.3%)	134 (45.4%)	
N previous pregnancies	0				
None (current first pregnancy)		69 (41.8%)	46 (35.4%)	115 (39.0%)	X^2^ (5)=4.9, *p* = .427
One		41 (24.8%)	43 (26.1%)	84 (28.5%)	
Two or more		55 (33.3%)	41 (31.5%)	96 (32.5%)	
N previous births	4				
None		49 (29.9%)	24 (18.9%)	73 (25.1%)	X^2^ (5)=6.1, *p* = .298
One		60 (36.6%)	56 (44.1%)	116 (39.9%)	
Two or more		55 (33.5%)	47 (37.0%)	102 (35.1%)	
Ethnic group	0				
White British		150 (90.9%)	128 (98.5%)	278 (94.2%)	X^2^ (8)=9.2, *p* = .329
White other		9 (5.5%)	1 (0.8%)	10 (3.4%)	
Asian/Black/Mixed		6 (3.6%)	1 (0.8%)	5 (1.7%)	
Other		2 (1.2%)	0 (0.0%)	2 (0.7%)	
Highest level of education	1				
Degree or higher degree		84 (50.9%)	75 (58.1%)	159 (54.1%)	X^2^ (6)=9.4, *p* = .154
No degree		81 (49.1%)	54 (41.9%)	135 (45.9%)	
Relationship status	0				
Married or living with partner		152 (92.1%)	121 (93.1%)	273 (92.5%)	X^2^ (2)=1.4, *p* = .500
Single or not living with partner		13 (7.9%)	9 (6.9%)	22 (7.5%)	
Employment	0				
In paid employment		129 (78.2%)	110 (84.6%)	239 (81.0%)	X^2^ (6)=8.3, *p* = .219
Not in paid employment		36 (21.8%)	20 (15.4%)	56 (19.0%)	
IMD decile					
1st tertile (1–6, most deprived)	0	62 (37.6%)	39 (30.0%)	101 (34.2%)	X^2^ (2)=3.4, *p* = .178
2nd tertile (7, 8)		53 (32.1%)	55 (42.3%)	108 (36.6%)	
3rd tertile (9–10, least deprived)		50 (30.3%)	36 (27.7%)	86 (29.2%)	

*p < .05; X^2^ – Based on the Chi-squared test; *IQR* Interquartile range (skewed data), *IMD* Index of Multiple Deprivation. Participants indicated their age group out of five groups, not their specific age. As most participants were in the age group 25–34 years, a division per quartile/tertile would have resulted in low number cells, so only two age categories are reported. For similar reasons, only three categories are presented for previous pregnancies/births, and for IMD decile. ‘Asian/Black/Mixed’ includes Asian British, Black British and Mixed White & Black Caribbean. ‘In paid employment’ includes those working full-time, part-time, as a free-lancer, or on maternity/sick leave from paid employment

The median IMD decile was 7 for the whole sample (IQR 3), corresponding to being in the 40% least deprived geographical area (technically known as a Lower Layer Super Output Area, or LSOA) in the country. In the urban group the number of women in each of the three IMD decile groups was roughly similar, but in the rural group a considerably larger proportion of women were in the second tertile, although differences were non-significant between the two groups. The characteristics of the participants are broadly representative of those in the region where the study took place with regard to average IMD score, ethnicity, female educational qualifications and employment rate [34] .

Table 2 provides data on access to services and social support. No significant differences existed between urban and rural groups with respect to any of these variables. Over a fifth (21.4%) had looked or asked for support or information about feelings of anxiety and/or depression during pregnancy or since the birth of their child. Amongst these women, partners were the most common reported source of support/information.

**Table 2:** Access to services and social support

Variable	Missing (n)	Urban (*N* = 165), n (%)	Rural (*N* = 130), n (%)	Overall (*N* = 295), n (%)	Differences between groups
Access to:					
GP	0	161 (97.6%)	128 (98.5%)	289 (98.0%)	X^2^ (1)=0.3, *p* = .593
Hospital	3	150 (92.0%)	113 (87.6%)	263 (90.1%)	X^2^ (1)=1.6, *p* = .209
Children’s Centre	2	130 (79.3%)	102 (79.1%)	232 (79.2%)	X^2^ (1)=0.0, *p* = .967
Health visitor	1	142 (86.1%)	116 (89.9%)	258 (87.8%)	X^2^ (1)=1.0, *p* = .316
Looked for support or information about feelings of anxiety and/or depression	0	38 (23.0%)	25 (19.2%)	63 (21.4%)	X^2^ (1)=0.6, *p* = .429
Sources of support/information about anxiety and/or depression (*n* = 63):					
Partner		28	17	45	X^2^ (1)=0.4, *p* = .506
Family		20	11	31	X^2^ (1)=0.6, *p* = .437
Websites		15	16	31	X^2^ (1)=3.3, *p* = .070
GP	1	20	9	29	X^2^ (1)=2.0, *p* = .162
Midwife		18	7	25	X^2^ (1)=2.6, *p* = .104
Friends		15	8	23	X^2^ (1)=0.5, *p* = .495
Health visitor		13	6	19	X^2^ (1)=0.9, *p* = .351
Mobile phone apps		3	3	6	–
Books or magazines		3	3	6	–
Other professional(s)		4	1	5	–
Other		0	0	0	–
		Med (IQR)	Med (IQR)	Med (IQR)	
Perceived social support – MSPSS:					
Subscale Significant Other	0	28 (3)	28 (4)	28 (3)	Z = 0.1, *p* = .930
Subscale Family	1	27 (4)	26 (4)	26 (4)	Z = 0.8, *p* = .430
Subscale Friends	0	24 (5)	25 (7)	25 (6)	Z = 0.9, *p* = .383
Total	1	78 (12)	77 (12)	78 (12)	Z = 0.9, *p* = .355

X^2^ – Based on the Chi-squared test; Z – Based on the Mann-Whitney test

Some difference tests were not performed due to the small number of observations

Sources of support/information: multiple responses allowed; from 63 participants who reported having sought support, so percentages are not presented to avoid confusion with full sample percentages

*MSPSS* Multidimensional Scale of Perceived Social Support. Subscale scores can range from 4 to 28; total scale scores can range from 12 to 84. Higher scores reflect stronger social support

Of a possible range of 4 to 28, perceived social support was highest with regards to a significant other (median 28, IQR 3), followed by support from family (median 26, IQR 4) and friends (median 25, IQR 6). Taking the sample overall, the median value for perceived social support was 78 (IQR 12), of a possible range of 12–84.

All models, across the three measures, showed a higher prevalence of mental illness in rural women (Table 3). In the unadjusted analysis (model 1), the prevalence of testing positive for depression and/or anxiety was 11.5% higher for rural compared to urban mothers, which was statistically significant (urban 28.5% vs rural 40.0%, *p* = .038). In all other models, differences though indicative were not statistically significant and confidence intervals were generally wide. For three of the four outcomes, adjusting for IMD and perinatal stage (model 2) was associated with a decrease in the odds ratio, which demonstrates that these variables explained some of the association as expected. For three of the four outcomes, the addition of social support (model 3) either led to increases in the odds ratio or to no change. The only exception was anxiety, where the odds ratio decreased slightly (from 1.24 to 1.16) in model 3, which indicates that social support had some explanatory value in the hypothesised direction.

**Table 3:** Odds of mental health illness in rural and urban women

Outcome variable	Urban (*N* = 165) n (%)	Rural (*N* = 130) n (%)	Overalln (%)	Logistic regression model	OR (SE) –Rural (cf urban)	95% CI	*p* value
Positive for depression and/or anxiety	47 (28.5%)	52 (40.0%)	99 (33.6%)	Model 1	1.67 (0.42)	1.03 to 2.72	.038*
				Model 2	1.57 (0.40)	0.95 to 2.58	.078
				Model 3	1.65 (0.46)	0.96 to 2.84	.070
Positive for depression - EPDS	30 (18.2%)	36 (27.7%)	66 (22.4%)	Model 1	1.72 (0.48)	0.99 to 2.99	.053
				Model 2	1.62 (0.48)	0.91 to 2.89	.100
				Model 3	1.69 (0.55)	0.90 to 3.19	.106
Positive for depression - Whooley	40 (24.2%)	44 (33.9%)	84 (28.5%)	Model 1	1.60 (0.41)	0.96 to 2.66	.071
				Model 2	1.54 (0.40)	0.92 to 2.58	.103
				Model 3	1.58 (0.45)	0.91 to 2.75	.105
Positive for anxiety - GAD-2	19 (11.5%)	18 (13.9%)	37 (12.5%)	Model 1	1.23 (0.43)	0.62 to 2.46	.549
				Model 2	1.24 (0.44)	0.61 to 2.50	.551
				Model 3	1.16 (0.45)	0.54 to 2.50	.696

*p < .05; OR - Odds ratio. SE – Standard error. Percentages correspond to unadjusted (raw) differences; denominator is N of rural/urban women. *N* = 295 for all models 1 & 2; *N* = 294 for all models 3

Model 1 – Association between rurality and outcome variable, unadjusted

Model 2 – Same as model 1, adjusted for IMD decile (by tertiles) and perinatal stage

Model 3 – Same as model 2, also adjusted for social support (MSPSS total score)

In an exploratory analysis, we compared women in the two most urban categories (*n* = 18) to those in the two most rural categories (*n* = 23) (Table 4). Only the descriptive data of this analysis are presented due to the small sample size. Both groups were comparable in most variables except perinatal stage, consistent with the full sample analysis. Although women in the most rural group experienced less socioeconomic deprivation, they were also more likely to look for support/information about depression/anxiety, and to report lower levels of social support especially from friends.

**Table 4:** Descriptive differences between the most urban and the most rural groups

Variable	Most urban group (*N* = 18)		Most rural group (*N* = 23)	
	n (%)	Median (IQR)	n (%)	Median (IQR)
Age, 25 years or above	17 (94.4%)		23 (100%)	
Perinatal stage				
Pregnant	15 (83.3%)		11 (47.8%)	
Postnatal	3 (16.7%)		12 (52.2%)	
Ethnicity – White British	18 (100%)		23 (100%)	
Education – Degree or higher	10 (55.6%)		17 (73.9%)	
Relationship – Married or living with partner	18 (100%)		23 (100%)	
Employment – In paid employment	16 (88.9%)		22 (95.7%)	
IMD deciles, by tertile:				
1st tertile (most deprived)	6 (33.3%)		6 (26.1%)	
2nd tertile	7 (38.9%)		12 (52.2%)	
3rd tertile (least deprived)	5 (27.8%)		5 (21.7%)	
Access to:				
GP	18 (100%)		23 (100%)	
Hospital	17 (94.4%)		22 (95.6%)	
Children’s centre	13 (72.2%)		18 (78.3%)	
Health visitor	13 (72.2%)		23 (100.0%)	
Has looked for support/information about depression/anxiety	1 (5.6%)		3 (13.0%)	
MSPSS Significant Other		28 (4)		28 (2)
MSPSS Family		28 (4)		27 (3)
MSPSS Friends		28 (4)		25 (7)
MSPSS total		84 (12)		79 (11)

*IMD* Index of Multiple Deprivation

*MSPSS* Multidimensional Scale of Perceived Social Support

*IQR* Interquartile Range

## Discussion

To our knowledge, this is the first quantitative study that compared mental health outcomes between perinatal women from rural and urban areas in the UK. Overall, our findings suggest that women in rural areas are at higher risk of perinatal depression and anxiety than their urban counterparts, with the odds ratio varying between 1.16 and 1.72 across outcomes. After accounting for potential confounding factors, though indicative, findings of a positive association between rurality and risk of perinatal mental illness did not reach statistical significance.

Rural and urban groups were comparable in all variables except perinatal stage; women in the urban group were more likely to be pregnant, whereas those in the rural group were more likely to have given birth already. This is likely to be due to the fact that health visitors who assisted us with recruitment happened to be more involved in rural areas and in postnatal visits at the time of the study. Even after adjusting for perinatal stage, our data revealed a trend, though non-statistically significant, for higher propensity for depression and anxiety in rural women. This suggests that other factors may exist that account for those differences. Some of those possible factors may not have been captured in our survey and include, as previously noted, greater expenditure with transport, poorer transport infrastructure and longer travel times [35], fewer options for childcare provision (other than a children’s centre) [36] or to attend antenatal and postnatal groups. Additional reasons may be a lack of stimulating activities and distance to amenities such as shops, church/temple or college [37], or to the gym, cinema or coffee shops, which can facilitate social interaction [38]. As measured in our study, (perceived) social support appeared to contribute little by way of explanation in the main analysis. However, our exploratory analysis suggested that rural-urban differences in social support exist, namely that women in very rural areas lack support from friends and, to lesser extent, from family, though this sub-analysis was based on a small sample.

The percentage of women at risk of anxiety in this study (11.5 to 13.9%) is similar to that reported in a nationally-representative study conducted in England [39]. However, the risk of depression appears to be unusually high in our study. Based on the Whooley test, 28.5% were positive screens, whereas on the EPDS, that number was 22.4%. Estimated rates of maternal mental illness range widely, depending on the assessment method, the timing of the assessment, and population characteristics. A literature review has reported the prevalence of maternal depression to be as high as 20% antenatally and between 12 to 16% postnatally [40], whereas the National Health Service (NHS) in England estimates both depression and anxiety rates to be 15 to 20% [1]. It is important to reiterate that none of the tools used in this study, including the EPDS (due to the choice of cut-off), aimed to assess rates of depression and anxiety as such, but rather, identify how many women would be at higher risk. For example, it is well-known that the Whooley is highly sensitive and should only be pre-diagnostic [22]. Likewise, the number of positive EPDS screens (22.4%) was higher than the previous figures reported above, and this may be due to various reasons:

i.) Lower cut-off score for postnatal women were used, as per existing guidelines [19]. However, this is not followed universally with many authors using the same cut off in the antenatal and postnatal periods [41].
ii.) Criterion of ‘at least probable minor depression’ (minimum score of 10 to 13), as opposed to ‘probable major depression’ (minimum score of 13 to 15) [19], which was intended to indicate how many women could be at risk, not to be diagnostic;
iii.) The timing of the postnatal assessments (children were on average 7 weeks old), as the health visitors who helped us with recruitment were more likely to be working with women at that early stage, which may been too soon to capture the decrease in depressive symptoms typically observed from pregnancy to after childbirth [42];
iv.) Women affected by mental illness may be more prone to take part in this type of research inflating overall estimates of rates of (or risk for) mental illness [43].

This study had several other limitations. The sample was relatively small and fell below our target sample of 348 which limited the power of our analysis. It consisted mainly of White-British women from an area of above average levels of affluence. As such, findings may not be generalisable to other parts of the UK or other countries. Although we assessed social support in various ways, we may still have missed some important aspects of this construct. The cross-sectional design of this study makes it impossible to make conclusions on causality. No information is available on the women who declined to take part. However, as discussed earlier, our sample compared well with the profile of the region where the study took place in all relevant sociodemographic variables [34], which suggests that no significant selection bias occurred.

The focus of this study was to compare rural and urban areas rather than estimating prevalence rates in each group. In addition, it is worth noting that both in the main and in the exploratory analysis more urban women lived in areas of higher socioeconomic deprivation (i.e., first tertile), although national data have shown that such geographical differences are not always clear-cut [35]. It is likely that financial and professional development support need to be part of efforts to increase the (mental) health of perinatal women in less affluent neighbourhoods, in addition to other forms of social support.

Future studies should aim to recruit a larger number of participants, ideally with matched rural and urban participants, to further explore whether those in rural areas are at greater risk of mental illness. This analysis will benefit from including participants in both ends of the spectrum, i.e., the most rural and most urban. Further quantitative and qualitative research is needed to investigate the contextual factors and potential mechanisms of such differences, e.g., role of friends, partners and extended family, or socioeconomic deprivation, to inform early intervention for mothers at risk of anxiety and depression.

## Conclusions

This study suggested that perinatal women from rural areas are at higher risk of depression and anxiety than their urban peers. Although this was not statistically significant when key confounding variables were considered, the direction of this association was consistent across analyses. More work is needed to replicate these findings in a larger sample in order to better understand the interplay between rurality, social deprivation and accessibility to support. This is necessary to identify and understand any inequalities that may exist between rural and urban areas in terms of antenatal and postnatal services, so that the right support can be provided where it is most needed, both to mothers and children at such a critical period of their lives.

## Supplementary information

**Additional file 1.** Questionnaire.

## Data Availability

The datasets used and/or analysed during the current study are available from the corresponding author on reasonable request.

## Abbreviations

EPDS: Edinburgh Postnatal Depression Scale
GAD-2: Generalized Anxiety Disorder 2-item
UK: United Kingdom
IMD: Index of Multiple Deprivation
MSPSS: Multidimensional Scale of Perceived Social Support
LSOA: Lower Layer Super Output Area
NHS: National Health Service
GP: General Practitioner
IQR: Interquartile Range
