# Supplementary material for: Rural-urban differences in the mental health of perinatal women: a UK-based cross-sectional study
Source: BMC Pregnancy Childbirth. 2020 Aug 14;20:464. doi: 10.1186/s12884-020-03132-2 (PMC7427846; doi:10.1186/s12884-020-03132-2)
Supplement: Supplementary file 1 — Additional file 1. Questionnaire. [file 12884_2020_3132_MOESM1_ESM.docx]

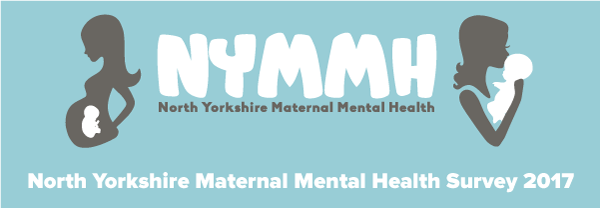
[references to the region where the study was conducted have been omitted]

- Over the next few months we are inviting women that are currently pregnant or have recently had a baby to take part in this short survey about their emotional health and wellbeing.
- The survey will take approximately 10 minutes to complete.
- Your answers, with those of other women, will be used to help us think about what we can do to best support women and families during pregnancy and the first year of their baby's life.
- Your responses are anonymized and confidential
- You can choose to be told whether or not your answers to Question 2 suggest you may be at risk of anxiety and/ or depression. If you have any concerns about your emotional health and wellbeing please contact your GP, Health Visitor or Midwife for advice and support.

*Before giving us your consent to take part please read the study information leaflet which has more information about the survey and why the research is being carried out.*

*Before completing the survey please complete the study consent form****.***

1*. Please tick which applies to you:*

- **I am currently expecting a baby**

…and I am ____________________ weeks pregnant.

- **I have recently had a baby**

…and my baby is ____________________ weeks old.

This section is about your emotional wellbeing.

2. *Please select the answer which comes closest to how you have felt in the PAST 7 DAYS, not just how you feel today.*

**I have been able to laugh and see the funny side of things**

- As much as I always could
- Not quite so much now
- Definitely not so much now
- Not at all

**I have looked forward with enjoyment to things**

- As much as I ever did
- Rather less than I used to
- Definitely less than I used to
- Hardly at all

**I have blamed myself unnecessarily when things went wrong**

- Yes, most of the time
- Yes, some of the time
- Not very often
- No, never

**I have been anxious or worried for no good reason**

- No, not at all
- Hardly ever
- Yes, sometimes
- Yes, very often

**I have felt scared or panicky for no good reason**

- Yes, quite a lot
- Yes, sometimes
- No, not much
- No, not at all

**Things have been getting on top of me**

- Yes, most of the time I haven't been able to cope at all
- Yes, sometimes I haven't been coping as well as usual
- No, most of the time I have coped quite well
- No, I have been coping as well as ever

**I have been so unhappy that I have had difficulty sleeping**

- Yes, most of the time
- Yes, some of the time
- Not very often
- No, not at all

**I have felt sad or miserable**

- Yes, most of the time
- Yes, some of the time
- Not very often
- No, not at all

**I have been so unhappy that I have been crying**

- Yes, most of the time
- Yes, quite often
- Only occasionally
- No, never

**The thought of harming myself has occurred to me**

- Yes, quite often
- Sometimes
- Hardly ever
- Never

3. *Please select either Yes or No to each of the following two questions:*

**During the past month have you often been bothered by feeling down, depressed or hopeless?**

- Yes
- No

**During the past month have you often been bothered by little interest or pleasure in doing things?**

- Yes
- No

4. **Over the past two weeks, how often have you been bothered by the following problems?**

*Please indicate how you feel about each statement by ticking one for each row only*

**Feeling anxious, nervous or on edge**

- Not at all
- Several days
- More than half the days
- Nearly every day

**Not being able to stop or control worrying**

- Not at all
- Several days
- More than half the days
- Nearly every day

This section is about support and access to services.

5. **We are interested in how you feel about the following statements.**

*Please read each statement carefully. Please indicate how you feel about each statement by ticking one for each row only.*

|  | **Very Strongly Agree** | **Strongly Agree** | **Mildly Agree** | **Neutral** | **Mildly Disagree** | **Strongly Disagree** | **Very Strongly Disagree** |
| --- | --- | --- | --- | --- | --- | --- | --- |
| There is a special person around when I am in need. |  |  |  |  |  |  |  |
| There is a special person with whom I can share my joys and sorrows. |  |  |  |  |  |  |  |
| My family really tries to help me. |  |  |  |  |  |  |  |
| I get the emotional help and support I need from my family. |  |  |  |  |  |  |  |
| I have a special person who is a real source of comfort to me. |  |  |  |  |  |  |  |
| My friends really try to help me. |  |  |  |  |  |  |  |
| I can count on my friends when things go wrong. |  |  |  |  |  |  |  |
| I can talk about my problems with my family. |  |  |  |  |  |  |  |
| I have friends with whom I can share my joys and sorrows. |  |  |  |  |  |  |  |
| There is a special person in my life who cares about my feelings. |  |  |  |  |  |  |  |
| My family is willing to help me make decisions. |  |  |  |  |  |  |  |
| I can talk about my problems with my friends. |  |  |  |  |  |  |  |

6. **Do you currently feel able to access the following services?** *Please tick either Yes or No for each of the services.*

|  | Yes | No |
| --- | --- | --- |
| **GP** |  |  |
| **Hospital** |  |  |
| **Children’s Centre** |  |  |
| **Health Visitor** |  |  |

*If you have ticked ‘Yes’ for all of the above, skip to question 8.*

7. **What have been the difficulties to accessing services?** (e.g. lack of transport, financial, feeling worried about how others will react)

8. **Have you looked for support/information about any feelings of anxiety and/or depression during your pregnancy or since the birth of your baby?**

- Yes
- No

*If ‘No’, please skip to question 10.*

9. **Where did you go to look for support/ information about any feelings of anxiety and/ or depression during your pregnancy or since the birth of your baby?**
*Please tick all that apply.*

- Partner
- Friends
- Family
- Midwife
- Health Visitor
- GP
- Other professional. *Please specify* ____________________
- Mobile phone Apps
- Websites
- Books or magazines
- Other. *Please specify* ____________________

This section is about you. (this information will be used to compare the responses of women with different circumstances, numbers of children, ages etc.)

10. **How many times have you been pregnant?**

- One
- Two
- Three
- Four
- Five
- More than five - *please state number* ____________________

11. **How many births have you had?**

____________________

12. **How old are you?**

- Under 18
- 18 - 24
- 25 - 34
- 35 - 44
- 45 - 54
- 55 - 64
- 65 or older

13. **What is your ethnic group?** *Please tick one box only.*

- White - British
- White - Irish
- Other White - European
- Other White. *Please Specify* ____________________
- Asian or Asian British - Pakistani
- Asian or Asian British - Bangladeshi
- Asian or Asian British - Indian
- Asian or Asian British - Chinese
- Asian or Asian British - Other. *Please Specify* ____________________
- Black or Black British - Caribbean
- Black or Black British - African
- Black or Black British - Other. *Please Specify* ____________________
- Mixed background - White and Black Caribbean
- Mixed background - White and Black African
- Mixed background - Other. *Please Specify* ____________________
- Any other ethnic group. *Please specify* ____________________
- I do not wish to say

14. **What is the highest level of education that you have reached?** *Please tick one box only.*

- I am still at school
- Left school before completing GCSEs
- GCSEs
- A levels/ Scottish Highers or International Baccalaureate
- Apprenticeship
- Professional qualifications
- First Degree (e.g. BSc, BA)
- Higher degree or above (e.g. MSc, MA, PhD)

15. **What best describes your current status?** *Please tick one box only.*

- Married or living with a partner
- Single
- Have a partner but not living together
- Other. *Please specify* ____________________

16. **What best describes your current employment?** *Please tick one box only.*

- In paid Full time employment
- In paid Part time employment
- Self-employed or freelance
- Studying or in training
- On Maternity Leave or Sick Leave from full time employment
- On Maternity Leave or Sick Leave from part time employment
- Not in paid employment

17. **What is your postcode?** (To compare the responses between people living in different areas)

____________________

This is the final section of the questionnaire.

18. **Please feel free to leave any additional comments in the box below relating to any of your answers so far.**

## Further Research

We would like to speak in more detail to women in [anonymized region] about their experiences during pregnancy and the first year of their baby's life. In particular about experiences of emotional and mental wellbeing during this period. This would involve an informal interview with the researcher at a time and place convenient to you.

19. **Are you interested in taking part in further research on the topic of mental health and wellbeing in pregnancy and early motherhood?** Participants will be offered a £10 high street voucher for taking part in the one to one interview.

- Yes, I would be interested in taking part in further research
- No, I would not be interested in taking part in further research

As a 'thank you', all women completing the survey can be entered into a prize draw to win one prize of £50 High Street Vouchers, which will be drawn within the next three months.

20. **Would you like to be entered into the prize draw?**  (Your contact details will be needed to notify you if you win, but will be separated from your questionnaire and destroyed after the study ends)

- Yes, I would like to be entered into the prize draw.
- No, I would not like to be entered into the prize draw.

21. **Would you like to be told whether your answers to Question 2 suggest you may be at risk of possible anxiety or depression?**  (Your contact details will be needed to notify you, but will be destroyed after the study ends)

- Yes, I would like to be told.
- No, I would not like to be told.

22. **What are your contact details?** (for further research and/or prize draw notification, or to be notified of your result)

Name

Telephone Number

Email address

If you have any concerns about your emotional health and wellbeing please contact your GP, Health Visitor or Midwife for advice and support.

**Thank you very much for completing the survey.**
